# Age and sex differentially shape brain networks in Parkinson's disease

**DOI:** 10.1111/cns.14149

**Published:** 2023-03-08

**Authors:** Zhichun Chen, Bin Wu, Guanglu Li, Liche Zhou, Lina Zhang, Jun Liu

**Affiliations:** ^1^ Department of Neurology and Institute of Neurology Ruijin Hospital Affiliated to Shanghai Jiao Tong University School of Medicine Shanghai China; ^2^ Department of Neurology The Second Xiangya Hospital Central South University Changsha China; ^3^ Department of Neurology Xuchang Central Hospital affiliated with Henan University of Science and Technology Henan China; ^4^ Department of Biostatistics Shanghai Jiao Tong University School of Medicine Shanghai China

**Keywords:** age, clinical phenotype, network, Parkinson's disease, sex

## Abstract

**Aims:**

Age and sex are important individual factors modifying the clinical symptoms of patients with Parkinson's disease (PD). Our goal is to evaluate the effects of age and sex on brain networks and clinical manifestations of PD patients.

**Methods:**

Parkinson's disease participants (*n* = 198) receiving functional magnetic resonance imaging from Parkinson's Progression Markers Initiative database were investigated. Participants were classified into lower quartile group (age rank: 0%~25%), interquartile group (age rank: 26%~75%), and upper quartile group (age rank: 76%~100%) according to their age quartiles to examine how age shapes brain network topology. The differences of brain network topological properties between male and female participants were also investigated.

**Results:**

Parkinson's disease patients in the upper quartile age group exhibited disrupted network topology of white matter networks and impaired integrity of white matter fibers compared to lower quartile age group. In contrast, sex preferentially shaped the small‐world topology of gray matter covariance network. Differential network metrics mediated the effects of age and sex on cognitive function of PD patients.

**Conclusion:**

Age and sex have diverse effects on brain structural networks and cognitive function of PD patients, highlighting their roles in the clinical management of PD.

## Graphical abstract

We found age specifically modified white matter network while sex preferentially affected gray matter covariance network. Additionally, we revealed different network metrics mediated the effects of age and sex on cognitive function of Parkinson's disease patients.

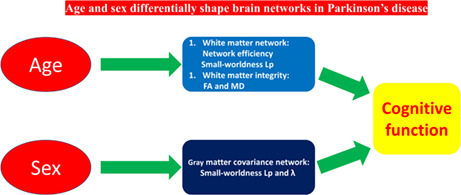

## 1 INTRODUCTION

Age and sex are essential demographic factors which significantly modify the clinical symptoms of patients with Parkinson's disease (PD).^1^, ^2^ Age is a major risk factor of PD and significantly associated with motor and non‐motor symptoms of PD patients.^2^, ^3^, ^4^ Additionally, striatum dopamine transporter binding measured by striatal binding ratios (SBRs) gradually decline as the patients' age increases.^4^ Sex is also associated with PD risk and male individuals have higher diagnosis probability of PD compared to females.^5^, ^6^ Compared to male PD patients, female PD patients generally exhibit milder motor symptoms, higher striatum SBRs, and relatively better cognitive function.^1^, ^2^, ^7^, ^8^ Therefore, age and sex play an important role in shaping the clinical manifestations of PD patients.

The significant impacts of age and sex on the clinical features of PD patients indicate that they affect brain networks of PD.^9^, ^10^, ^11^ Actually, a previous study showed that age is associated with more white matter hyperintensities in PD patients,^11^ indicating age may modify white matter networks of PD patients. In contrast, sex seems to affect gray matter structure and gray matter covariance networks.^9^, ^12^ Tremblay et al.^12^ revealed that male PD patients have greater gray matter atrophy in 11 cortical regions while female patients have greater atrophy in 6 cortical regions. Additionally, a previous study showed that male PD patients exhibit significant reductions of cortical thickness in multiple cortical regions compared to female patients.^9^ Thus, it is likely that age specifically affects white matter networks while sex preferentially modifies gray matter covariance networks in PD. According to previous literature, how age and sex shape structural and functional networks of PD patients is poorly understood. Additionally, how the network metrics modified by age and sex are causally correlated with clinical features of PD patients is also unclear.

In this study, our hypothesis is that age and sex affect clinical manifestations of PD patients through the modifications of brain networks. In consequence, we have two major objectives in current study: (i) assess how age and sex affect the functional network, white matter network, and gray matter covariance network of PD patients. (ii) examine whether brain networks contribute to the effects of age and sex on clinical manifestations of PD patients. To evaluate how age shapes brain networks, we used two methods. In the first method, given that age is a continuous variable, we divided the PD patients into 3 age groups according to their age quartiles: lower quartile age group (Q1, age rank: 0%~25%), interquartile age group (Q2–3, age rank: 26%~75%), and upper quartile age group (Q4, age rank: 76%~100%), which helped us to quantitatively assess whether higher age group exhibited different clinical features and network metrics compared to lower age groups. In the second method, we used multivariate regression analysis to examine the associations between age and clinical assessments or network metrics with sex, years of education, and disease duration as covariates. The second method can help us to validate the results revealed by the first method and test whether the effects of age on clinical assessments or network metrics are independent of other confounding variables. Similarly, we used comparisons of group difference between male and female patients and multivariate regression analysis to evaluate the effects of sex on clinical assessments or network metrics of PD patients. Finally, the mediation analysis was utilized to examine whether network metrics mediated the associations between age or sex and clinical features in PD patients.

## 2 METHODS

### 2.1 Study population

The raw data used in this study were obtained from Parkinson's Progression Markers Initiative (PPMI) database which is sponsored by Michael J Fox Foundation. The Institutional Review Board of all the participating sites approved the PPMI study and the written informed consents of participants can be obtained from the site investigators. For up‐to‐date PPMI data information, visit www.ppmi‐info.org. PD patients met the inclusion criteria below: (i) The participant was diagnosed to have PD according to current diagnostic criteria^13^; (ii) The participants acquired 3D T1‐weighted MPRAGE imaging, resting‐state fMRI imaging, and diffusion tensor imaging (DTI) during the same period; (iii) The participant showed normal brain structure in both T1‐weighted and T2‐weighted MRI images; (iv) they received the evaluations of motor symptoms, non‐motor symptoms, CSF indices, and striatum SBRs. PD subjects were excluded if they had a clinical diagnosis of dementia or other neuropsychiatric diseases except PD. The inclusion criteria for control participants (*n* = 189) were shown below: (i) they were required to be age 30 years, or older; (ii) they should receive the clinical assessments that PD patients examined, including striatum SBRs; (iii) they were relatively healthy and had no systemic diseases that may affect their neurological assessments. The control participants were excluded if they met the exclusion criteria shown below: (i) they were diagnosed with an active, clinically significant neurological disorder; (ii) they had first‐degree relative with PD; (iii) they carried genetic mutations of PD demonstrated by whole exome sequencing; Both control and PD participants were excluded if they were treated with neuroleptics, metoclopramide, α‐methyldopa, methylphenidate, reserpine, or amphetamine derivative or were currently treated with anticoagulants. Both PD and control subjects were not from the genetic PPMI cohort and prodromal cohort. Because most of control participants had no MRI images and thus their MRI images were not analyzed. According to above inclusion criteria, totally 198 PD participants were included for the final analysis. All of them (*n* = 198) acquired 3D T1 images whereas only 146 of them performed DTI imaging (*n* = 146) and 83 of 146 patients with DTI images received resting‐state fMRI (*n* = 83). The demographic and clinical assessments of control and PD patients have been compared and posted online in Appendix S1 of a preprint of another study.^14^ Count densities for each region (caudate, putamen, and striatum) were extracted from dopaminergic imaging with 123I Ioflupane targeting the dopamine transporter (DAT‐SPECT) and used to calculate SBRs for each of the striatal regions. The SBRs were calculated as (target region/reference region)‐1.^15^ The reference region was the occipital lobe. The associations between age or sex and clinical assessments of PD patients were shown in Table 1. The levodopa equivalent daily dose (LEDD) data were not available in most PD patients; thus, they were not included in the analysis. To assess the effects of age on brain networks and clinical assessments, PD patients were divided into lower quartile age group (Q1, age rank: 0%~25%, age range: 33.72~54.35), interquartile age group (Q2–3, age rank: 26%~75%, age range: 54.56~68.96), and upper quartile age group (Q4, age rank: 76%~100%, age range: 68.98~82.25) based on their age quartiles. The demographic and clinical data of subjects included for each subgroup division were shown in Table S1, which is included in the Appendix S1. The similar approach of subgroup divisions according to age quartiles has been described previously.^16^, ^17^

**TABLE 1:** Associations between age, sex, and clinical assessments.

Motor assessments				
	HY	Tremor	Rigidity	UPDRS‐III
Age	*β* = 0.01, *p* < 0.01	*β* = 0.07, *p* < 0.05	*β* = 0.01, *p* > 0.05	*β* = 0.20, *p* < 0.01
Sex	*β* = −0.05, *p* > 0.05	*β* = 0.21, *p* > 0.05	*β* = −1.10, *p* < 0.01	*β* = −2.19, *p* > 0.05

Non‐motor assessments								
	RBDSQ	SCOPA‐AUT (*n* = 192/187)	LNS	BJLOT	SFT	SDMT	MoCA (*n* = 197/189)	HVLT‐R Immediate Recall
Age	*β* = −0.003, *p* > 0.05	*β* = 0.14 *p* < 0.01	*β* = −0.12, *p* < 0.0001	*β* = −0.06, *p* < 0.0001	*β* = −0.23, *p* < 0.01	*β* = −0.36, *p* < 0.0001	*β* = −0.07, *p* < 0.001	*β* = −0.16, *p* < 0.0001
Sex	*β* = −1.04, *p* < 0.05	*β* = −0.08, *p* > 0.05	*β* = 0.08, *p* > 0.05	*β* = −1.50, *p* < 0.0001	*β* = 5.18, *p* < 0.01	*β* = 4.42, *p* < 0.01	*β* = 0.39, *p* > 0.05	*β* = 3.03, *p* < 0.0001

Striatum SBR (*n* = 193/186)								
	Caudate_R	Caudate_L	Putamen_R	Putamen_L	Striatum_R	Striatum_L	Bilateral Caudate	Bilateral putamen
Age	*β* = −0.01 *p* < 0.01	*β* = −0.01 *p* < 0.01	*β* = −0.005 *p* < 0.05	*β* = −0.007 *p* < 0.01	*β* = −0.02 *p* < 0.01	*β* = −0.02 *p* < 0.01	*β* = −0.01 *p* < 0.01	*r* = −0.006 *p* < 0.01
Sex	*β* = 0.14 *p* > 0.05	*β* = 0.26 *p* < 0.01	*β* = 0.07 *p* > 0.05	*β* = 0.15 *p* < 0.01	*β* = 0.21 *p* > 0.05	*β* = 0.41 *p* < 0.01	*β* = 0.20 *p* < 0.05	*β* = 0.11 *p* < 0.01

CSF indices				
	Aβ level (pg/mL) (*n* = 176/181)	α‐syn level (pg/mL) (*n* = 178/183)	Tau level (pg/mL) (*n* = 172/180)	p‐tau level (pg/mL) (*n* = 159/170)
Age	*β* = −4.20 *p* > 0.05	*β* = 6.10 *p* > 0.05	*β* = 0.82 *p* > 0.05	*β* = 0.10 *p* < 0.05
Sex	*β* = 33.52 *p* > 0.05	*β* = 155.3 *p* > 0.05	*β* = 9.38 *p* > 0.05	*β* = −0.06 *p* > 0.05

*Note*: The data were shown as the *β* and *p* values derived from multivariate regression analysis with age, sex, years of education, and disease duration as covariates. The motor function examination was assessed in ON state.

Abbreviations: Aβ, β‐amyloid; BJLOT, Benton Judgment of Line Orientation; CSF, cerebrospinal fluid; HVLT‐R, Hopkins Verbal Learning Test – Revised; HY, Hoehn & Yahr stage; LNS, Letter Number Sequencing; MoCA, Montreal Cognitive Assessment; RBDSQ, REM Sleep Behavior Disorder Screening Questionnaire; SBR, striatal binding ratio; SCOPA‐AUT, Scale for Outcomes in Parkinson's Disease‐Autonomic; SDMT, Symbol Digit Modalities Test; SFT, Semantic Fluency Test Score; UPDRS‐III, Unified Parkinson’ s Disease Rating Scale Part III; α‐syn, α‐synuclein.

### 2.2 Image acquisition

All the fMRI images were obtained on 3 Tesla machines (either Trio™ or Verio™ systems, Siemens Healthcare). The parameters for 3D T1 MPRAGE sequence are: TR = 2300 ms, TI = 900 ms, TE = 2.98 ms, Voxel size = 1 mm^3^, Slice thickness = 1.2 mm, twofold acceleration, sagittal‐oblique angulation. The resting‐state fMRI images were acquired using gradient‐echo T2*‐weighted echo planar imaging (EPI) sequence. The parameters for resting‐state fMRI are: TR = 2400 ms, TE = 25 ms, Voxel size = 3.3 mm^3^, Slice thickness = 3.3 mm, Slice number = 40, Acquisition time = 8 min, Flip angle = 90°. Subjects were required to rest quietly, with their eyes open and not to fall asleep. The parameters for DTI sequence are: TR = 8400–8800 ms, TE = 88 ms, Voxel size = 2 mm^3^, Slice thickness = 2 mm, Slice number = 72, twofold acceleration, axial‐oblique aligned along the anterior–posterior commissure, 64 directions, b = 1000 s/mm^2^ along with diffusion‐unweighted b0 images.

### 2.3 Imaging preprocessing

Before formal data preprocessing, the MRI images in DICOM format were converted into NIFTI format using dcm2nii in the MRIcroN software (http://www.mccauslandcenter.sc.edu/mricro/mricron/).

#### 2.3.1 3D T1 imaging preprocessing

The T1 images (*n* = 198) for voxel‐based morphometry (VBM) were processed using VBM8 toolbox implemented in Statistical Parametric Mapping 12 software (SPM12, https://www.fil.ion.ucl.ac.uk/spm/software/spm12/). The T1 images were initially normalized with Diffeomorphic Anatomic Registration Trough Exponentiated Lie algebra (DARTEL) template, and then segmented into gray matter, white matter, and cerebrospinal fluid (CSF). The segmented gray matter images were smoothed before the construction of gray matter covariance network.

#### 2.3.2 Diffusion tensor imaging preprocessing

The DTI data (*n* = 146) were preprocessed using FMRIB Software Library toolbox (FSL, https://fsl.fmrib.ox.ac.uk/fsl/fslwiki). Initially, the brain skull was stripped and brain was individually extracted using Brain Extraction Tool (BET). To correct the head motion and eddy‐current distortions, we performed eddy‐current correction by applying a rigid‐body transformation to the b0 image using the eddy_correct command of FSL. Then, the diffusion tensor metrics were calculated and individual fractional anisotropy (FA), mean diffusivity (MD), axial diffusivity, and radial diffusivity maps were derived. To do this, dtifit command of FSL was utilized. Finally, individual images were normalized into standard MNI space for comparisons across participants.

#### 2.3.3 Resting‐state imaging preprocessing

For magnetization equilibrium, the first 10 volumes of individual functional images (*n* = 83) were generally discarded. Slice timing correction was performed to temporally align all slices to a reference time‐point. Then the functional images were realigned to register individual images to the first volume of the data and spatially normalized using standard EPI template. Spatial smoothing was performed using a 4‐mm Gaussian filter to improve the signal‐to‐noise ratio and attenuate anatomical variances. To reduce the effects of non‐neuronal fluctuations, the head motion profiles (Friston 24 parameters), the CSF signal, and the white matter signals were regressed out. Finally, the functional images were band‐pass filtered to reduce the effects of low‐frequency drift and high‐frequency physiological noises. The frequency band for temporal filtering is 0.01 to 0.1 HZ.

To adjust the effects of head motions on functional images, we excluded participants with frame‐wise displacement (FD) of head motions >0.5 mm and head rotation >2°.^18^, ^19^, ^20^, ^21^ According to this criteria, nine participants were excluded from functional network analysis due to the head motions. In addition, we also regressed out the head motion parameters to adjust the effects of head motions on functional image. Furthermore, we compared the group difference of head motion power FD and no statistical significance was observed (Table S2).

### 2.4 Network construction

#### 2.4.1 Gray matter covariance network construction

After T1 imaging preprocessing, the individual gray matter covariance network was extracted using the methodology proposed by Tijms et al.^22^ Briefly, the method defines network nodes as 3 x 3 x 3 voxel cubes, and the network edge as correlation coefficient of gray matter morphology between each pair of nodes. We normalized gray matter networks using Automated Anatomical Labeling (AAL) parcellation atlas, which created a 90 x 90 gray matter covariance matrix for individual subject using a previously published method.^23^, ^24^ To improve the normality of gray matter covariance edge, we performed Fisher's *r*‐to‐*z* transformation of the correlation coefficients.

#### 2.4.2 White matter network construction

We implemented deterministic fiber tractography to construct the white matter network matrix using a free open Matlab toolbox PANDA (http://www.nitrc.org/projects/panda/). The Fiber Assignment by Continuous Tracking (FACT) algorithm was utilized to create the whole‐brain white matter fibers between each pair of 90 cortical and subcortical nodes from AAL atlas. The FA threshold is set to 0.2 and angle threshold is set to 45°. After the tractography, a white matter network based on fiber number (FN) was constructed for individual subject.

#### 2.4.3 Functional network construction

We constructed individual interregional functional connectivity matrix in two major steps. The first step is to use AAL atlas to define the 90 cortical and subcortical nodes. The second step is to compute pairwise functional connectivity with linear Pearson's correlation of time series from 90 nodes. To improve the normality of functional connectivity, we performed Fisher's *r*‐to‐*z* transformation of correlation coefficients. This will create a 90 x 90 functional connectivity matrix for each subject.

### 2.5 Graph‐based network analysis

We used GRETNA (https://www.nitrc.org/projects/gretna/) to calculate topological properties of three types of networks.^25^ A wide range of network sparsity thresholds (0.05~0.50 with an interval of 0.05) were set to compute the global network properties in each type of network, including assortativity, hierarchy, synchronization, global efficiency, local efficiency, and small‐worldness properties: clustering coefficient (Cp), characteristic path length (Lp), normalized clustering coefficient (*γ*), normalized characteristic path length (*λ*), and small worldness (*σ*). The area under curve (AUC) was also calculated for each network metric which was independent of any single sparsity threshold. Detailed definitions of each network metric can be found in previous studies.^26^, ^27^

### 2.6 Statistical analysis

The normality of clinical and imaging metric data was performed with Shapiro–Wilk test and Anderson‐Darling test. Data that do not meet a normal/Gaussian distribution were analyzed using non‐parametric test.

#### 2.6.1 Comparison of clinical assessments

The group differences of clinical assessments among different age quartiles in both control and PD groups were evaluated by ANOVA test followed by Tukey's post‐hoc test. The group differences of clinical assessments between male and female participants in both control and PD groups were also evaluated by ANOVA test followed by Tukey's post‐hoc test. *p* < 0.05 was considered statistically significant.

#### 2.6.2 Comparison of global network metrics

For the comparison of global network metrics at each sparsity threshold, we used two‐way ANOVA test followed by false discovery rate (FDR) correction.^28^ *p* < 0.05 for each sparsity threshold was considered statistically significant. The AUCs of global networks were compared with two‐sample unpaired *t*‐test for two group comparison or one‐way ANOVA test for three group comparison, which were followed by FDR correction. *p* < 0.05 was considered statistically significant.

#### 2.6.3 Comparisons of DTI metrics

Because the white matter network of the brain is composed of a large number of white matter tracts, the integrity of white matter tracts directly affects the topology of structural network. To further demonstrate that age specifically shapes white matter structure but not gray matter structure, the effects of age or sex on FA and MD values extracted from common white matter tracts were also assessed in our study, which would make our study more complete and solid. To compare DTI metrics (FA or MD) of multiple white matter tracts among three age quartile groups, two‐way ANOVA test followed by FDR correction was used. The same statistic test was also used for the comparisons of DTI metrics between male patients and female patients. During the ANOVA test, two separate FDR corrections were applied to FA and MD measures, respectively. *p* < 0.05 was considered statistically significant.

#### 2.6.4 Association analysis between clinical assessments and age or sex

The associations between age and clinical assessments were examined by multivariate regression analysis with sex, disease duration, and years of education as covariates. The associations between sex (1 = Male, 2 = Female) and clinical assessments were examined by multivariate regression analysis with age, disease duration, and years of education as covariates. *p* < 0.05 was considered statistically significant.

#### 2.6.5 Association analysis between imaging metrics and age or sex

The associations between age and graphical or DTI metrics were examined by multivariate regression analysis with sex, disease duration, and years of education as covariates. The associations between sex and network or DTI metrics were examined by multivariate regression analysis with age, disease duration, and years of education as covariates. *p* < 0.05 was considered statistically significant.

#### 2.6.6 Mediation analysis

The mediation analysis was performed using IBM SPSS Statistics 20. The independent variable in the mediation model was age or sex. The dependent variables include both cognitive assessments and non‐cognitive assessments. The mediators were brain network metrics or DTI metrics. We modeled the mediated relationships (indirect path) between age or sex and clinical assessments. The model also included the direct path from age or sex to the clinical assessments of PD patients. During the mediation analysis, age, sex, years of education, and disease duration were selectively included as covariates. *p* < 0.05 was considered statistically significant.

## 3 RESULTS

### 3.1 The effects of age and sex on clinical assessments

**FIGURE 1:**
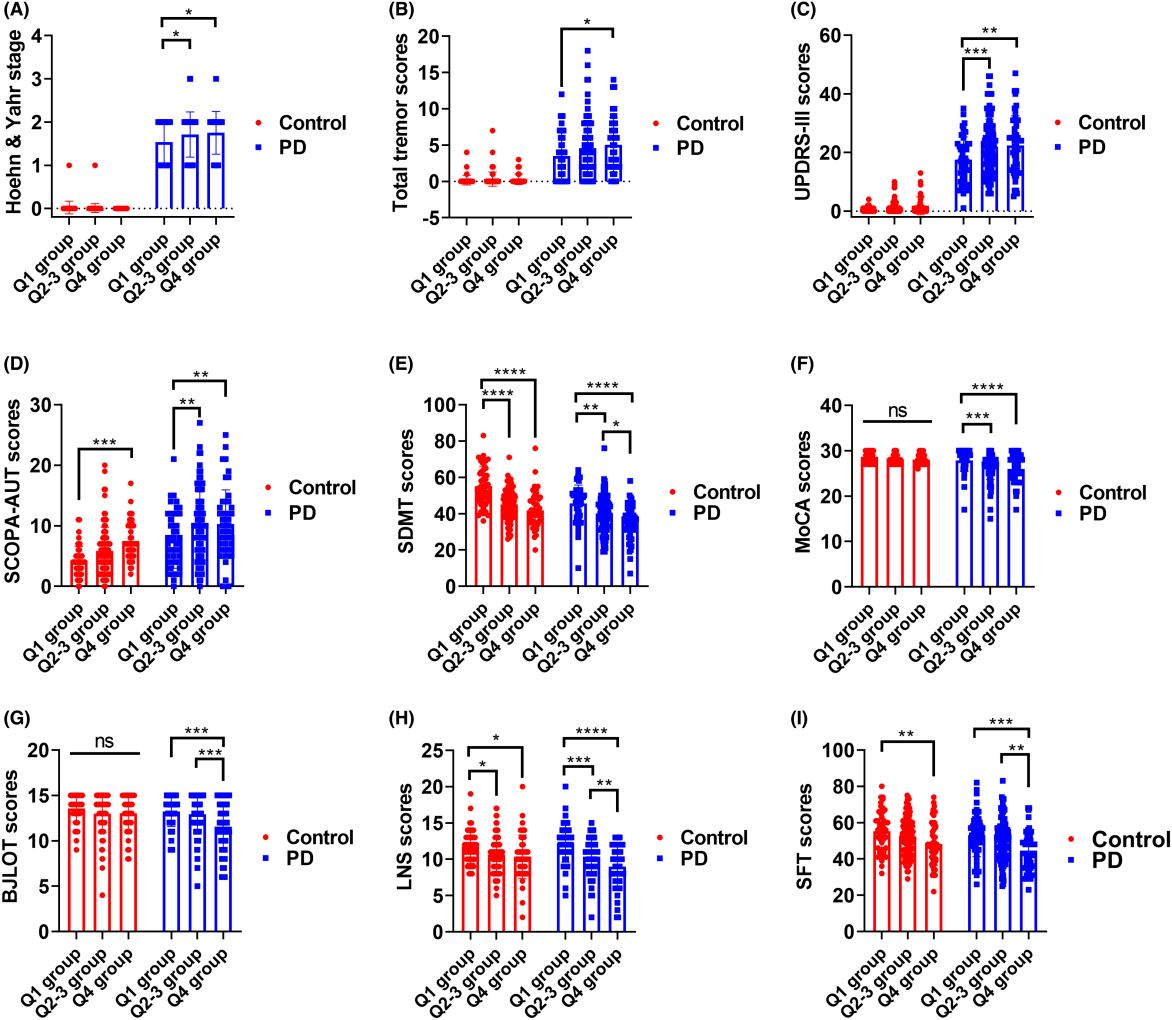
Group differences of clinical assessments among different age quartiles of control and PD participants. Compared to PD patients in Q1 group (*n* = 49), PD patients in Q4 group (*n* = 50) showed higher Hoehn & Yahr stages (A), tremor scores (B), UPDRS‐III scores (C), SCOPA‐AUT scores (D) and lower SDMT scores (E), MoCA scores (F), BJLOT scores (G), LNS scores (H), and SFT scores (I). Compared to PD patients in Q1 group (*n* = 49), PD patients in Q2–3 group (*n* = 99) also had higher Hoehn & Yahr stages (A), UPDRS‐III scores (C), SCOPA‐AUT scores (D) and lower SDMT scores (E), MoCA scores (F), and LNS scores (H). Two‐way ANOVA test followed by Tukey's post‐hoc test was used for the comparisons of clinical assessments among different age quartiles of PD patients. *p < 0.05, **p < 0.01, ***p < 0.001, ****p < 0.0001. Abbreviations: BJLOT, Benton Judgment of Line Orientation; LNS, Letter Number Sequencing; MoCA, Montreal Cognitive Assessment; SCOPA‐AUT, Scale for Outcomes in Parkinson's Disease‐Autonomic; SDMT, Symbol Digit Modalities Test; SFT, Semantic Fluency Test Score; UPDRS‐III, Unified Parkinson’ s Disease Rating Scale Part III.

The demographic and clinical features of both control and PD patients have been compared and presented online in Appendix S1 of a preprint of another study^14^ and thus were not included in current study. The effects of age on clinical assessments were shown in Figure 1. Compared to PD patients in Q1 group (*n* = 49, age range: 33.72~54.35), PD patients in Q4 group (*n* = 50, age range: 68.98~82.25) exhibited higher Hoehn & Yahr stages (*p* < 0.05), tremor scores (*p* < 0.05), Unified Parkinson's Disease Rating Scale Part III (UPDRS‐III) scores (*p* < 0.01), Scale for Outcomes in Parkinson's Disease‐Autonomic (SCOPA‐AUT) scores (*p* < 0.01) and lower Symbol Digit Modalities Test (SDMT) scores (*p* < 0.0001), Montreal Cognitive Assessment (MoCA) scores (*p* < 0.0001), Benton Judgment of Line Orientation (BJLOT) scores (*p* < 0.001), Letter Number Sequencing (LNS) scores (*p* < 0.0001), and Semantic Fluency Test (SFT) scores (*p* < 0.001). Compared to PD patients in Q1 group (*n* = 49), PD patients in Q2–3 group (*n* = 99, age range: 54.56~68.96) also had higher Hoehn & Yahr stages (*p* < 0.05), UPDRS‐III scores (*p* < 0.001), SCOPA‐AUT scores (*p* < 0.01) and lower SDMT scores (*p* < 0.01), MoCA scores (*p* < 0.001), and LNS scores (*p* < 0.001).

We also evaluated the effects of age on SBRs of striatal regions in PD patients (Figure S1). Compared to PD patients in Q1 group, PD patients in Q4 group showed lower SBRs in right caudate (*p* < 0.01; Figure S1A), left caudate (*p* < 0.01; Figure S1B), left putamen (*p* < 0.05; Figure S1D), right striatum (*p* < 0.01; Figure S1E), left striatum (*p* < 0.01; Figure S1F), bilateral caudate (*p* < 0.001; Figure S1G), bilateral putamen (*p* = 0.055; Figure S1H), and bilateral striatum (*p* < 0.01; Figure S1I). Compared to PD patients in Q1 group (Figure S1), PD patients in Q2–3 group also exhibited lower SBRs in right caudate (*p* < 0.01), left caudate (*p* < 0.001), left putamen (*p* < 0.01), right striatum (*p* < 0.01), left striatum (*p* < 0.001), bilateral caudate (*p* < 0.001), bilateral putamen (*p* < 0.05), and bilateral striatum (*p* < 0.01).

For the effects of sex on clinical assessments of PD patients, we found female PD patients (*n* = 74) showed lower UPDRS‐III scores (*p* < 0.05), rigidity scores (*p* < 0.0001), BJLOT scores (*p* < 0.0001) and higher scores of SDMT (*p* < 0.05), SFT (*p* < 0.01), Immediate Recall of Hopkins Verbal Learning Test – Revised (HVLT‐R; (*p* < 0.0001) compared to male patients (*n* = 124, Figure S2A‐E). In addition, female PD patients also exhibited higher SBRs in bilateral caudate (*p* < 0.05; Figure S2F) and striatum (*p* < 0.05; Figure S2H).

### 3.2 Associations between clinical assessments and age or sex

In 198 participants from PPMI database, after adjusting sex, years of education, and disease duration, age was significantly associated with motor symptoms (Hoehn & Yahr stages, Tremor scores, UPDRS‐III scores), non‐motor symptoms (scores of SCOPA‐AUT, LNS, BJLOT, SDMT, SFT, MoCA, and Immediate Recall of HVLT‐R), striatum SBRs, and p‐tau levels in CSF of PD patients (Table 1). In contrast, sex (1 = Male, 2 = Female) was independently associated with rigidity score (*β* = −1.10, *p* < 0.01), REM Sleep Behavior Disorder Screening Questionnaire (RBDSQ) score (*β* = −1.04, *p* < 0.05), BJLOT score (*β* = −1.50, *p* < 0.0001), SDMT score (*β* = 4.42, *p* < 0.01), SFT score (*β* = 5.18, *p* < 0.01), Immediate Recall of HVLT‐R (*β* = 3.03, *p* < 0.0001), and striatum SBRs after the adjustment of age, years of education, and disease duration (Table 1).

### 3.3 Age, sex, and topology of brain networks

**FIGURE 2:**
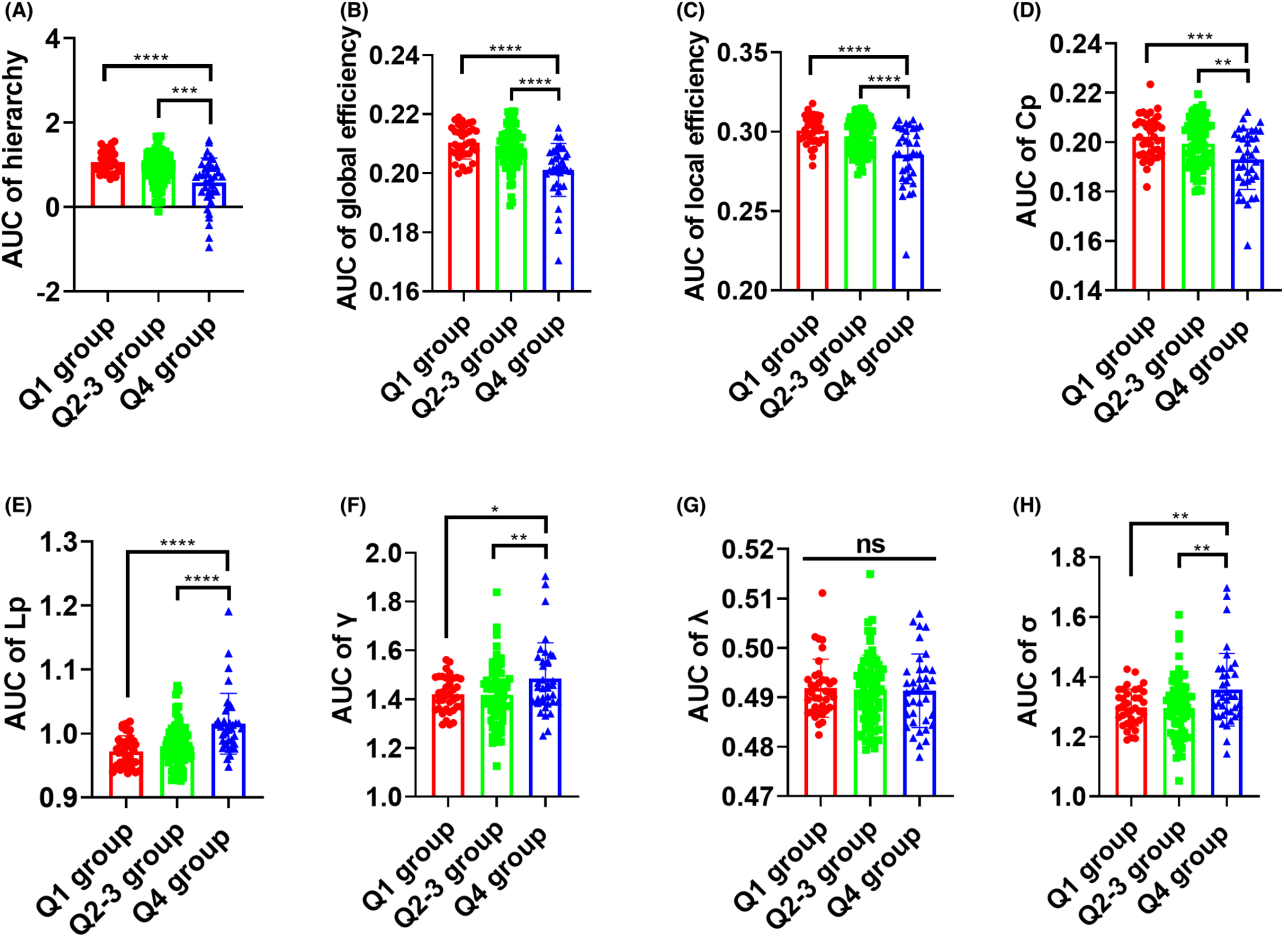
Group differences in the AUCs of white matter network metrics among different age quartiles of PD patients. Group differences in the AUC of hierarchy (A), AUC of global efficiency (B), AUC of local efficiency (C), AUC of Cp (D), AUC of Lp (E), AUC of *γ* (F), AUC of *λ* (G), and AUC of *σ* (H). ANOVA test followed by FDR correction was used for the comparisons of AUCs of graphical metrics among three age quartile groups. *p < 0.05, **p < 0.01, ***p < 0.001, ****p < 0.0001. Abbreviations: AUC, area under curve; Cp, clustering coefficient; Lp, characteristic path length.

The global network metrics of functional network and gray matter covariance network were not significantly different among three age quartile groups (*p* > 0.05, data not shown). The network assortativity and synchronization in white matter network was also not significantly different among three age groups. However, compared to PD patients in Q1 group (*n* = 36) and Q2–3 group (*n* = 73), the PD patients in Q4 group (*n* = 37) exhibited reduced AUCs of hierarchy, global efficiency, local efficiency, small‐worldness Cp and increased AUCs of small‐worldness Lp, small‐worldness *γ*, and small‐worldness *σ* in white matter network (Figure 2). Consistently, compared to PD patients in Q1 group and Q2–3 group, PD patients in Q4 group also showed reductions of hierarchy, global efficiency, local efficiency, small‐worldness Cp and elevations of small‐worldness Lp, small‐worldness *γ*, and small‐worldness *σ* at multiple network sparsity threshold in white matter network (Figure S3).

There was no significant difference in the global network metrics of white matter network and functional network between male and female PD patients (*p* > 0.05, data not shown). However, compared to male patients (*n* = 124), female patients (*n* = 74) exhibited increased AUCs of small‐worldness Lp (*p* < 0.01, FDR corrected) and *λ* (*p* < 0.01, FDR corrected) while the AUC of small‐worldness Cp, *γ*, and *σ* were not statistically different (Figure S4).

To independently assess the effects of age on network metrics of PD patients, we examined the associations between age and network metrics using multivariate regression analysis including sex, years of education and disease duration as covariates. As shown in Table 2 (*n* = 146), age was significantly associated with AUCs of hierarchy (*β* = −0.019, *p* < 0.0001), global efficiency (*β* = −0.0004, *p* < 0.0001), local efficiency (*β* = −0.0006, *p* < 0.0001), small‐worldness Cp (*β* = −0.0004, *p* < 0.0001), small‐worldness Lp (*β* = 0.002, *p* < 0.0001), small‐worldness *γ* (*β* = 0.003, *p* < 0.05), and small‐worldness *σ* (*β* = 0.002, *p* < 0.01), which is independent of sex, years of education, and disease duration.

**TABLE 2:** Associations between age, sex, and graphical metrics of white matter network.

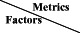	Assortativity	Hierarchy	Synchronization	Global efficiency
Age	*β* = 0.007 *p* > 0.05	*β* = −0.019 *p* < 0.0001	*β* = 0.01 *p* > 0.05	*β* = −0.0004 *p* < 0.0001
Sex	*β* = −0.17 *p* > 0.05	*β* = −0.06 *p* > 0.05	*β* = 0.05 *p* > 0.05	*β* = −0.001 *p* > 0.05

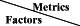	Local efficiency	Small‐worldness Cp	Small‐worldness Lp	Small‐worldness *γ*
Age	*β* = −0.0006 *p* < 0.0001	*β* = −0.0004 *p* < 0.0001	*β* = 0.002 *p* < 0.0001	*β* = 0.003 *p* < 0.05
Sex	*β* = −0.004 *p* > 0.05	*β* = −0.002 *p* > 0.05	*β* = 0.006 *p* > 0.05	*β* = −0.007 *p* > 0.05

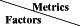	Small‐worldness *λ*	Small‐worldness *σ*
Age	*β* = −3.141 e‐005 *p* > 0.05	*β* = 0.002 *p* < 0.01
Sex	*β* = −0.001 *p* > 0.05	*β* = −0.002 *p* > 0.05

The data were shown as the *β* and *p* values derived from multivariate regression analysis including age, sex, years of education, and disease duration as covariates.

Abbreviations: Cp, clustering coefficient; Lp, characteristic path length; *γ*, normalized clustering coefficient; *λ*, normalized characteristic path length; *σ*, small worldness.

Similarly, multivariate regression analysis showed that sex (1 = Male, 2 = Female) was significantly associated with AUCs of small‐worldness Lp (*β* = 0.014, *p* < 0.01) and *λ* (*β* = 0.004, *p* < 0.01), but not associated with AUCs of small‐worldness Cp (*β* = 0.0009, *p* = 0.08), *γ* (*p* > 0.05), and *σ* (*p* > 0.05), indicating female patients have higher AUCs of small‐worldness Lp and *λ*.

### 3.4 Age, sex, and white matter integrity

**FIGURE 3:**
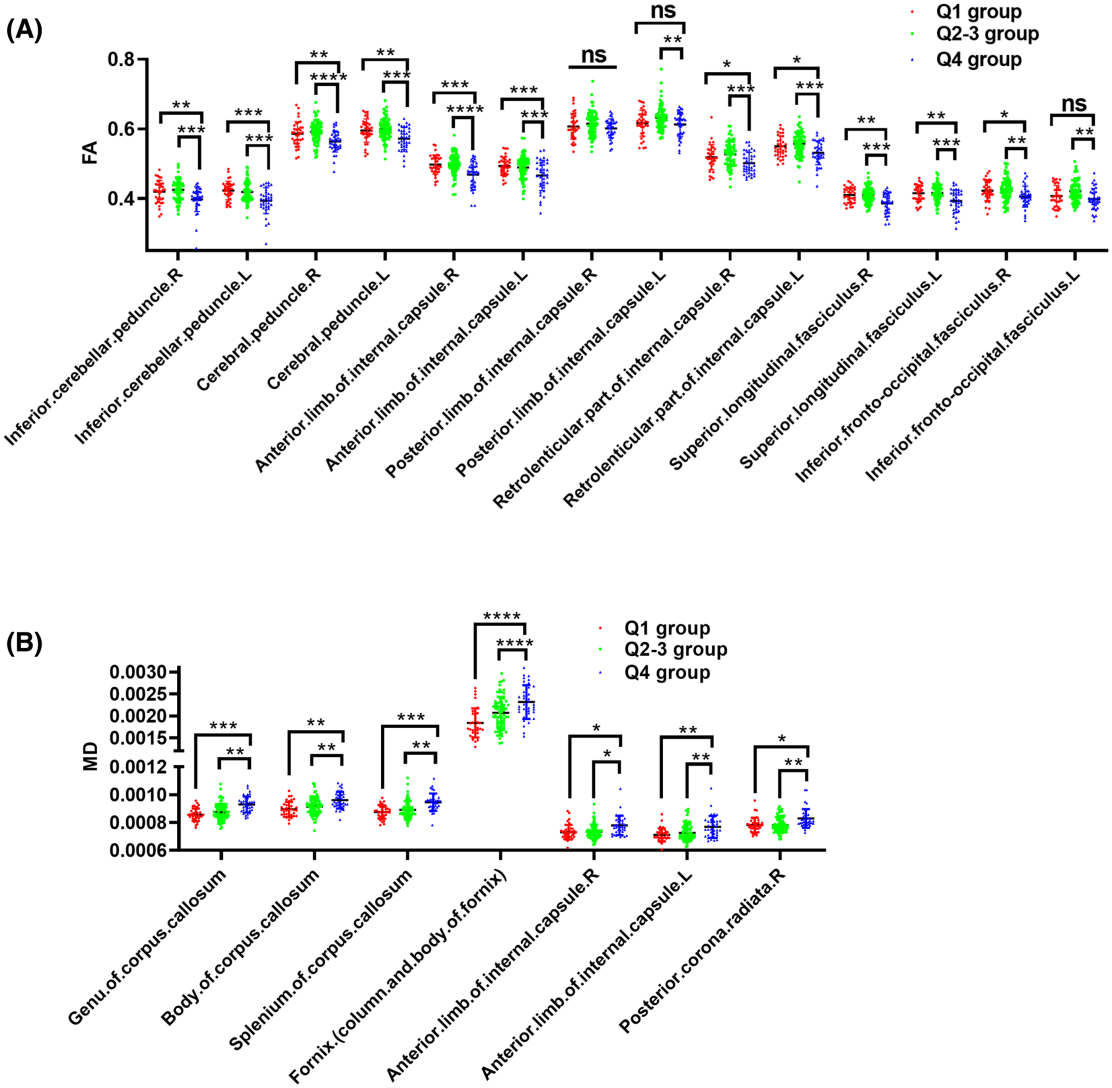
Group differences of DTI metrics among different age quartiles of PD patients. Group differences of FA (A) and MD (B). Two‐way ANOVA test followed by FDR correction was used for the comparisons of FA and MD among three age quartile groups. *p < 0.05, **p < 0.01, ***p < 0.001, ****p < 0.0001. Abbreviations: DTI, Diffusion tensor imaging; FA, Fractional anisotropy; MD, Mean diffusivity.

The changes of topology of white matter network suggested that white matter integrity was also significantly different among three age quartile groups. Compared to PD patients in Q1 and Q2–3 group, PD patients in Q4 group had significantly reduced FA in multiple white matter tracts, including bilateral inferior cerebellar peduncle, bilateral cerebral peduncle, bilateral anterior limb of internal capsule, bilateral retrolenticular part of internal capsule, and bilateral superior longitudinal fasciculus (all *p* < 0.05, FDR corrected; Figure 3A). In addition, PD patients in Q4 group showed increased MD in corpus callosum, fornix, and bilateral anterior limb of internal capsule compared to Q1 and Q2–3 group (all *p* < 0.05, FDR corrected; Figure 3B). It should be noted that only FA and MD measures of partial white matter tracts were shown in Figure 3.

There were no significant differences in DTI metrics between male and female PD patients (*p* > 0.05, FDR corrected, data not shown).

The multivariate regression analysis showed that age was significantly associated with the DTI metrics (FA and MD) in multiple white matter tracts, such as corpus callosum, fornix, bilateral inferior cerebellar peduncle, bilateral cerebral peduncle, bilateral anterior limb of internal capsule, and bilateral anterior corona radiata (Tables 3 and 4), which is independent of sex, years of education, and disease duration.

**TABLE 3:** Associations between age and FA of white matter tracts.

White matter tracts	*β* values	*p* values	Corrected *p* values
Genu.of.corpus.callosum	**−0.002**	**<0.0001**	**<0.0001**
Body.of.corpus.callosum	**−0.001**	**<0.0001**	**<0.0001**
Splenium.of.corpus.callosum	**−0.001**	**=0.0001**	**<0.001**
Fornix.(column.and.body.of.fornix)	**−0.004**	**<0.0001**	**<0.0001**
Corticospinal.tract.R	−0.0001	>0.05	>0.05
Corticospinal.tract.L	−0.00006	>0.05	>0.05
Inferior.cerebellar.peduncle.R	**−0.0008**	**<0.01**	**<0.05**
Inferior.cerebellar.peduncle.L	**−0.0009**	**<0.01**	**<0.01**
Superior.cerebellar.peduncle.R	0.0002	>0.05	>0.05
Superior.cerebellar.peduncle.L	0.00009	>0.05	>0.05
Cerebral.peduncle.R	**−0.0008**	**<0.05**	**<0.05**
Cerebral.peduncle.L	**−0.0007**	**<0.05**	**<0.05**
Anterior.limb.of.internal.capsule.R	**−0.001**	**<0.01**	**<0.01**
Anterior.limb.of.internal.capsule.L	**−0.001**	**<0.01**	**<0.01**
Posterior.limb.of.internal.capsule.R	−0.0001	>0.05	>0.05
Posterior.limb.of.internal.capsule.L	−0.0001	>0.05	>0.05
Retrolenticular.part.of.internal.capsule.R	−0.0007	<0.05	>0.05
Retrolenticular.part.of.internal.capsule.L	−0.0006	<0.05	>0.05
Anterior.corona.radiata.R	**−0.001**	**<0.0001**	**<0.0001**
Anterior.corona.radiata.L	**−0.001**	**<0.0001**	**<0.0001**
Superior.corona.radiata.R	−0.0005	>0.05	>0.05
Superior.corona.radiata.L	−0.0003	>0.05	>0.05
Posterior.corona.radiata.R	**−0.0006**	**<0.05**	**<0.05**
Posterior.corona.radiata.L	−0.0006	<0.05	>0.05
Posterior.thalamic.radiation.(include.optic.radiation).R	**−0.001**	**<0.001**	**<0.001**
Posterior.thalamic.radiation.(include.optic.radiation).L	**−0.001**	**<0.01**	**<0.01**
Superior.longitudinal.fasciculus.R	**−0.0007**	**<0.01**	**<0.01**
Superior.longitudinal.fasciculus.L	**−0.0006**	**<0.05**	**<0.05**
Inferior.fronto‐occipital.fasciculus.R	**−0.0007**	**<0.05**	**<0.05**
Inferior.fronto‐occipital.fasciculus.L	−0.0004	>0.05	>0.05
Uncinate.fasciculus.R	0.000006	>0.05	>0.05
Uncinate.fasciculus.L	−0.0004	>0.05	>0.05
Tapetum.R	−0.0007	>0.05	>0.05
Tapetum.L	**−0.0009**	**<0.05**	**<0.05**

*Note*: The data were shown as the *β*, *p*, and FDR‐corrected *p* values derived from multivariate regression analysis including sex, years of education, and disease duration as covariates.

Abbreviations: FA, fractional anisotropy.

The bold values indicate statistical significance after FDR correction.

**TABLE 4:** Associations between age and MD of white matter tracts.

White matter tracts	*β* values	*p* values	Corrected *p* values
Genu.of.corpus.callosum	**0.000003**	**<0.0001**	**<0.0001**
Body.of.corpus.callosum	**0.000003**	**<0.0001**	**<0.0001**
Splenium.of.corpus.callosum	**0.000003**	**<0.0001**	**<0.0001**
Fornix.(column.and.body.of.fornix)	**0.00002**	**<0.0001**	**<0.0001**
Corticospinal.tract.R	0.000001	>0.05	>0.05
Corticospinal.tract.L	0.0000006	>0.05	>0.05
Inferior.cerebellar.peduncle.R	**0.000001**	**<0.05**	**<0.05**
Inferior.cerebellar.peduncle.L	**0.000001**	**<0.05**	**<0.05**
Superior.cerebellar.peduncle.R	0.0000001	>0.05	>0.05
Superior.cerebellar.peduncle.L	0.0000005	>0.05	>0.05
Cerebral.peduncle.R	**0.000001**	**<0.05**	**<0.05**
Cerebral.peduncle.L	**0.000001**	**<0.05**	**<0.05**
Anterior.limb.of.internal.capsule.R	**0.000002**	**<0.001**	**<0.001**
Anterior.limb.of.internal.capsule.L	**0.00008**	**<0.0001**	**<0.001**
Posterior.limb.of.internal.capsule.R	0.0000003	>0.05	>0.05
Posterior.limb.of.internal.capsule.L	0.0000001	>0.05	>0.05
Retrolenticular.part.of.internal.capsule.R	**0.000001**	**<0.01**	**<0.01**
Retrolenticular.part.of.internal.capsule.L	**0.000001**	**<0.01**	**<0.05**
Anterior.corona.radiata.R	**0.000002**	**<0.001**	**<0.01**
Anterior.corona.radiata.L	**0.000002**	**<0.0001**	**<0.0001**
Superior.corona.radiata.R	**0.000001**	**<0.01**	**<0.01**
Superior.corona.radiata.L	**0.000002**	**<0.001**	**<0.01**
Posterior.corona.radiata.R	**0.000002**	**<0.001**	**<0.01**
Posterior.corona.radiata.L	**0.000001**	**<0.01**	**<0.01**
Posterior.thalamic.radiation.(include.optic.radiation).R	**0.000001**	**<0.05**	**<0.05**
Posterior.thalamic.radiation.(include.optic.radiation).L	**0.000001**	**<0.01**	**<0.01**
Superior.longitudinal.fasciculus.R	**0.000001**	**<0.001**	**<0.001**
Superior.longitudinal.fasciculus.L	**0.000001**	**<0.01**	**<0.01**
Inferior.fronto‐occipital.fasciculus.R	**0.000002**	**<0.001**	**<0.01**
Inferior.fronto‐occipital.fasciculus.L	**0.000002**	**<0.0001**	**<0.0001**
Uncinate.fasciculus.R	0.0000006	>0.05	>0.05
Uncinate.fasciculus.L	**0.000002**	**<0.001**	**<0.01**
Tapetum.R	**0.000006**	**<0.0001**	**<0.0001**
Tapetum.L	**0.000009**	**<0.0001**	**<0.0001**

*Note*: The data were shown as the *β*, *p*, and FDR‐corrected *p* values derived from multivariate regression analysis including sex, years of education, and disease duration as covariates. The bold values indicate statistical significance after FDR correction.

Abbreviations: MD, mean diffusivity.

The bold values indicate statistical significance after FDR correction.

### 3.5 Mediation analysis

**FIGURE 4:**
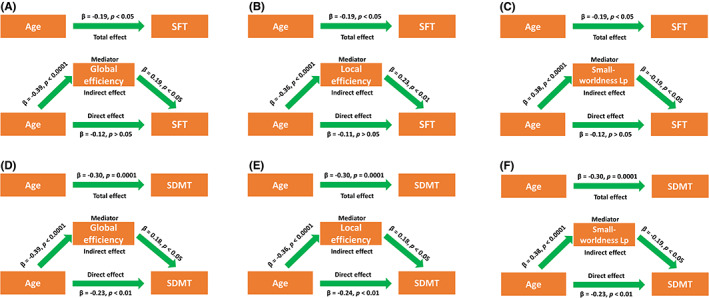
The white matter network topology mediated the relationship between age and cognitive assessments of PD patients. Mediation analysis of global efficiency and SFT (A), local efficiency and SFT (B), small‐worldness Lp and SFT(C), global efficiency and SDMT (D), local efficiency and SDMT (E), and small‐worldness Lp and SDMT (F). During the mediation analysis, sex, years of education, and disease duration were included as covariates. *p* < 0.05 was considered statistically significant. Abbreviations: Lp, characteristic path length; SDMT, Symbol Digit Modalities Test; SFT, Semantic Fluency Test Score.

The global network metrics of white matter network, including global efficiency, local efficiency, and small‐worldness Lp mediated the negative relationship between age and SFT scores of PD patients (Figure 4A‐C). The global network metrics of white matter network, such as global efficiency, local efficiency, and small‐worldness Lp, partially mediated the negative relationship between age and SDMT scores of patients (Figure 4D‐F).

**FIGURE 5:**
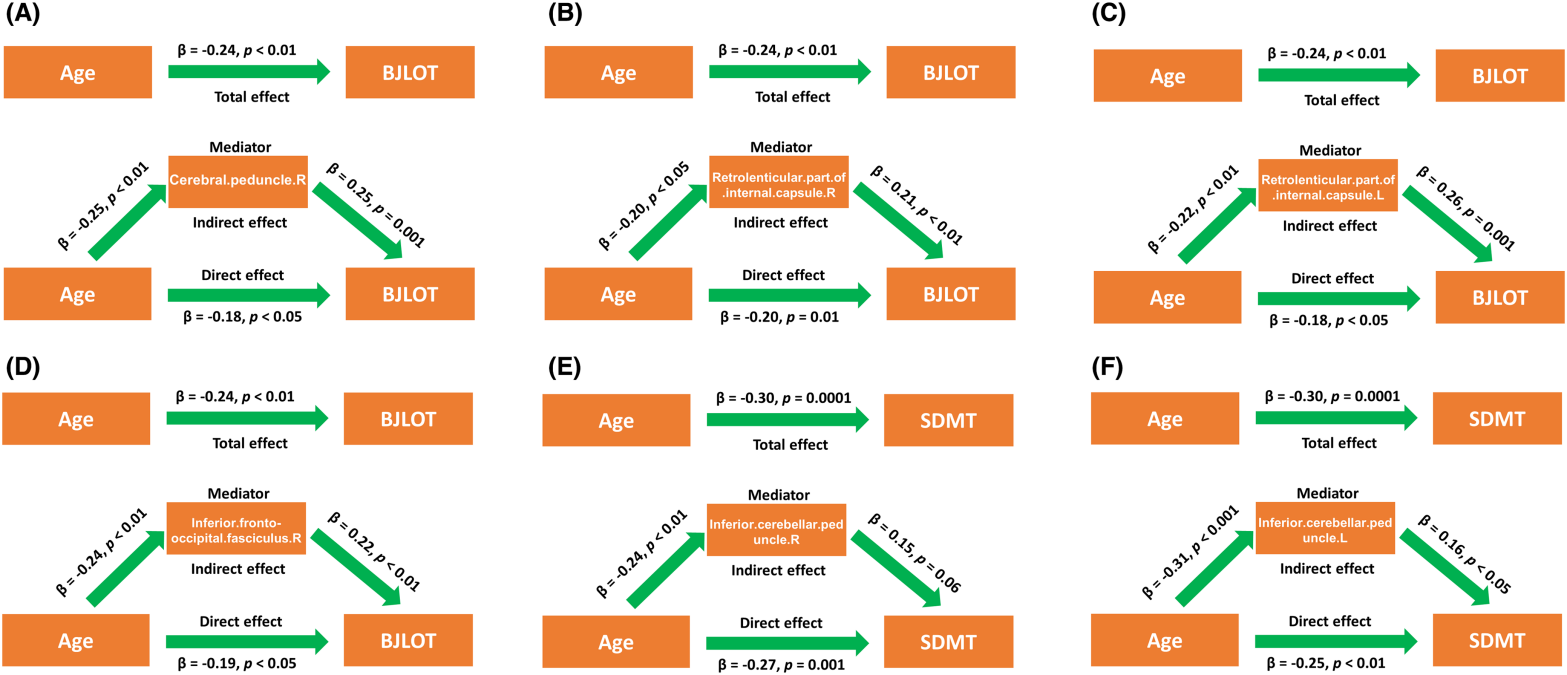
The integrity of white matter tracts mediated the relationship between age and cognitive assessments of PD patients. Mediation analysis of right cerebral peduncle and BJLOT (A), retrolenticular part of right internal capsule and BJLOT (B), retrolenticular part of left internal capsule and BJLOT (C), right inferior frontal‐occipital fasciculus and BJLOT (D), right inferior cerebellar peduncle and SDMT (E), and left inferior cerebellar peduncle and SDMT (F). During the mediation analysis, sex, years of education, and disease duration were included as covariates. *p* < 0.05 was considered statistically significant. Abbreviations: BJLOT, Benton Judgment of Line Orientation; SDMT, Symbol Digit Modalities Test.

The FA of white matter tracts, including right cerebral peduncle, right retrolenticular part of internal capsule, left retrolenticular part of internal capsule, and right inferior frontal‐occipital fasciculus, partially mediated the negative relationship between age and BJLOT scores of PD patients (Figure 5A‐D). The FA of bilateral inferior cerebellar peduncle partially mediated the negative relationship between age and SDMT scores of PD patients (Figure 5 E,F).

The network metrics of gray matter covariance network also showed significant mediation effects. As shown in Figure S5, the AUC of small‐worldness Lp and *λ* partially mediated the negative relationship between sex and BJLOT scores of PD patients (Figure S5A,B).

## 4 DISCUSSION

In this study, we evaluated the effects of age and sex on brain networks of PD patients and found age specifically modified white matter network while sex preferentially affected gray matter covariance network. Additionally, we revealed different network metrics mediated the effects of age and sex on the clinical assessments of PD patients.

We replicated previous findings that age and sex are significantly associated with clinical manifestations of PD patients.^1^, ^2^, ^4^, ^7^ As age increases, the motor symptoms and non‐motor symptoms become more and more severe, and striatum SBRs gradually decline.^2^, ^4^ In current study, we revealed the motor symptoms of male PD patients were much worse than female patients, which was consistent with previous studies showing that female patients exhibit milder motor impairment compared to male patients.^8^, ^29^ Similar to previous studies,^30^ we revealed the female patients had better verbal memory and language function than male patients. Whereas we found visuospatial judgment evaluated by BJLOT was much worse in female patients compared to male patients, which was consistent with previous longitudinal study showing that female patients exhibit greater decline in visuospatial domain.^31^ Though SDMT scores were higher in female patients, the MoCA scores were not significantly different between male and female patients, which was consistent with the result reported by a recent study.^30^ We also replicated previous findings that female patients have higher striatum SBRs than male patients,^4^, ^32^ which supported the notion that female patients have less nigra‐striatal impairment and motor symptoms. It deserved to be noted that the effects of age and sex on clinical manifestations were independent of disease duration, which is an important confounding variable in the multivariate regression analysis. One limitation of our study is that LEDD data were not included as a covariate in the multivariate regression analysis, given that it was considered during the association analysis of a previous study.^33^ However, it seemed that LEDD was not associated with cognitive function in PD patients without dementia.^34^

Because age significantly modified the clinical features of PD patients, we explored how age shaped brain networks of PD patients. We found age had no significant effects on the global network metrics of functional network and gray matter covariance network. These findings suggested that age was not associated with prominent topological reorganization of functional network and gray matter covariance network in PD. We found age specifically shaped white matter network topology and white matter integrity in PD patients, which was consistent with a recent study showing aging is associated with a higher load of white matter lesions in PD.^11^ Due to the lack of DTI images in control group, whether these age‐associated alterations were specific to PD or occurred in both healthy control and PD participants remained unknown. According to a previous study, age was dramatically associated with white matter microstructure in healthy people aged 44.64–77.12 years from the UK Biobank.^35^ Similarly, in a longitudinal multi‐site diffusion MRI study of 1218 healthy participants, researchers found age was negatively correlated with anisotropies and positively correlated with diffusivities in multiple white matter tracts.^36^ Based on these findings from heathy control participants and our data in Tables 3 and 4, it is possible that age‐related alterations in DTI metrics in PD overlap with those changes in heathy participants. Future studies will be required to understand whether the age‐related changes in white matter structure are different between healthy elderly and PD patients.

We found age‐associated changes in white matter network metrics were causally correlated with the cognitive assessments of PD patients, implying increased age contributes to cognitive impairment in PD by shaping white matter network. Consistently, Scamarcia et al.^37^ demonstrated that the volume of white matter hyperintensities changed over time and was significantly associated with impairment of global cognition, executive functions, and language in PD. They also found that baseline volume of white matter hyperintensities was a moderate risk factor for progression to mild cognitive impairment.^37^ Similarly, another study reported that periventricular white matter lesions correlated with decline in executive functions and working memory in PD and PD patients with cognitive impairment.^38^ These findings and our results suggested that age‐associated changes in white matter structure partially mediated cognitive decline in PD patients. Notably, we found no significant correlations between UPDRS‐III scores and topological or DTI metrics of white matter network, indicating that age‐associated changes in white matter network may not contribute to motor manifestations of PD. This notion was supported by a recent longitudinal study showing that DTI measures did not vary significantly as disease progressed and DTI measures were not significantly associated with clinical deterioration of motor symptoms.^37^ However, we found HY stage was correlated with DTI metrics although UPDRS‐III scores were not correlated with topological or DTI metrics of white matter network. For example, we found HY stage was significantly associated with FA of left retrolenticular part of internal capsule (*β* = −0.22, *p* < 0.01) and the mediation analysis supported that FA of left retrolenticular part of internal capsule mediated the effects of age on HY stage (*β* = −0.17, *p* < 0.05). Taken together, our study provides evidence that white matter structural metrics assessed by MRI are an important tool for monitoring both cognitive impairment and motor deterioration in PD patients.

Aging is the major risk factor for PD and contributes to the initiation and progression of PD.^39^ However, the neural mechanisms underlying the effects of aging on neurodegeneration of PD were still unknown. In current study, we found aging had significant impacts on white matter networks of PD patients, which may provide potential mechanisms to explain how aging contributes to neurodegeneration in PD. According to previous literature, PD patients exhibited widespread impairment of white matter integrity^37^, ^40^ and white matter degeneration was associated with faster progressive parkinsonism in PD.^41^ In addition, white matter hyperintensity burden was a reliable predictor of cognitive decline in PD patients.^42^, ^43^, ^44^ Therefore, aging may shape the progression of motor and non‐motor symptoms in PD by inducing white matter damages.^37^ In our study, we provide evidence that aging shapes both clinical manifestations and white matter networks of PD patients. Our findings have important implications in both preclinical and clinical conditions. Most of previous studies used young animals to establish PD models, however, young animals are more resistant to PD‐related pathophysiology than old animals.^45^ Therefore, animal models that better characterize clinical disease within the elderly would be more beneficial, as it will increase the translational value and attenuate the risk of failures in clinical trials.^45^ In fact, Miller et al.^46^ have ever found both aging and genetic susceptibility were required for the occurrence of disease phenotypes, such as loss of tyrosine hydroxylase (TH) expression and enlarged mitochondria or Lewy‐body‐precursor inclusions using a premature aging model by expressing progerin, a truncated form of lamin A. Because aging plays an essential role in the pathogenesis of PD, anti‐aging therapies would provide new opportunities for the development of disease‐modifying treatments for PD.^47^, ^48^ A previous study revealed that anti‐aging protein klotho alleviated the injury of dopaminergic neurons in 6‐hydroxydopamine (6‐OHDA) rat model of PD, which was involved in PKA/CaMKII/CREB signaling.^47^ Another study found that anti‐aging treatments can slow aggregate propagation of α‐synuclein by restoring lysosomal function.^48^ In our study, we found aging specifically modified the white matter network in PD, which contributes to the cognitive impairment of PD patients. Thus, future study is required to demonstrate whether age‐related white matter destruction contribute to PD pathogenesis and examine whether improving white matter integrity is a potential therapy to rescue age‐associated cognitive deficits in PD.^43^, ^49^

In current study, we revealed age specifically shaped white matter network with no effects on gray matter covariance network. Some previous literature has suggested that PD patients exhibited both gray matter atrophy and white matter degeneration and axonal degeneration may lead to gray matter atrophy,^50^, ^51^ thus, it is likely that age may also affect gray matter structure by induction of white matter degeneration. Additionally, the effects of age on cortical thickness have been described previously.^52^, ^53^ Nevertheless, because the major purpose of our study was to evaluate the effects of age on brain network metrics, the effects of age on cortical thickness of PD patients were not studied. Future studies were required to investigate whether age affected cortical thickness and gray matter volume in PD patients.

We found sex had no significant effects on the global network metrics of white matter network and functional network. In contrast, sex significantly modified the small‐world topology of gray matter covariance network. Consistently, previous studies have shown that sex modifies gray matter structure and gray matter covariance network of PD patients,^9^, ^12^ characterized by significantly different atrophy patterns and network topology between male and female patients. Oltra et al.^54^ reported that male PD patients exhibited cortical thinning in postcentral and precentral regions and greater atrophy in cortical and subcortical regions, including thalamus, caudate, putamen, and brainstem, compared to female PD patients. Interestingly, another study revealed that males had greater atrophy in both cortical and subcortical regions than females in PD patients with rapid eye movement sleep behavior disorder.^55^ Our results of increased small‐worldness properties in female patients were consistent with previous findings based on gray matter covariance network constructed by cortical thickness.^9^ Therefore, we conclude that small‐worldness topology in gray matter covariance network is significantly modified by sex in PD. Importantly, we found small‐worldness properties mediated the effects of sex on cognitive function of PD patients. These results are novel and provide evidence that sex‐related changes in small‐world topology partially explained the variations of cognitive function in PD patients.

The mechanisms underlying sex‐dependent variations in clinical manifestations and gray matter structure in PD are still largely unknown. The sex hormones may play a role in the shaping of episodic memory by sex in PD. Conner et al.^56^ (2020) found circulating gonadal hormones modulated the negative impacts of 6‐OHDA on episodic‐like memory and androgen deficiency induced by gonadectomy provided powerful and selective protections against the harmful effects of 6‐OHDA on memory functions in male rats. Aside from the detrimental actions of sex hormones in males, emerging evidence suggested that sex‐chromosome genes may also contribute to the male bias of disease risk and sex‐specific differences in clinical manifestations of PD.^57^ It has been known that Y‐chromosome gene, *SRY*, directly modulates brain function in males, which is independent of sex hormone.^57^ Reducing nigral *SRY* expression in male rats attenuated motor deficits and dopaminergic neurodegeneration in 6‐OHDA‐induced and rotenone‐induced rat PD models through the inhibition of DNA damage, mitochondrial degradation, and neuroinflammation.^57^ In a tissue‐specific meta‐analysis, researches detected 15 genes with sex‐differential patterns in PD, which were involved in mitochondrial function, oxidative stress, neuronal degeneration, and cell death.^58^ These findings indicate that multiple molecular pathways may be associated with sex‐specific variations in clinical features and gray matter structure of PD. Future studies are required to explore the mechanisms mediating the effects of sex on brain networks and cognitive function in PD. Taken together, our study suggests that sex‐associated differences in both clinical manifestations and gray matter structure are nonnegligible in PD and should be considered in future clinical and translational research.^59^

We found age or sex had different effects on functional and structural networks. In fact, previous studies have shown that PD patients exhibited divergent patterns of changes in functional and structural network.^60^, ^61^, ^62^, ^63^, ^64^, ^65^ Moreover, due to different computation methodologies, functional network and structural network were not usually consistent,^66^, ^67^ but they provided complementary understanding about the changes of brain structure and function.^68^ Furthermore, the results from our and other studies all suggested age or sex had differential impacts on different imaging metrics.^52^, ^53^, ^54^, ^55^, ^63^, ^69^ To conclude, age and sex may exert diverse effects on different network types of PD patients.

## 5 CONCLUSIONS

In this study, we found that age and sex contribute to the heterogeneity of clinical manifestations of PD patients in a network‐dependent manner. These findings suggest that the effects of age and sex should be considered for the complete understanding of disease pathogenesis and optimal management of PD patients.

## AUTHOR CONTRIBUTIONS

JL and ZCC: conceptualization (lead), writing—original draft (lead), formal analysis (lead), writing—review and editing (equal). BW and GLL: data curation (lead), formal analysis (supporting), writing—review and editing (supporting). LCZ and LNZ: writing—review and editing (supporting). All authors contributed to the article and approved the submitted version.

## FUNDING INFORMATION

This work was supported by grants from the National Key Research and Development Program (2016YFC1306505), the National Natural Science Foundation of China (81471287, 81071024, 81171202). These funding sources had no role in study design, conduct, analysis, interpretation or writing of the manuscript, or in the decision to submit the manuscript.

## CONFLICT OF INTEREST STATEMENT

The authors declare that they have no conflicts of interest.

## PATIENT CONSENT STATEMENT

The written informed consents of participants can be obtained from the participating site investigators of PPMI study.

## Supporting information


Appendix S1

## Data Availability

The data that support the findings of this study are available from the corresponding author upon reasonable request.
