# Supplementary material for: Age and sex differentially shape brain networks in Parkinson's disease
Source: CNS Neurosci Ther. 2023 Mar 8;29(7):1907–22. doi: 10.1111/cns.14149 (PMC10324368; doi:10.1111/cns.14149)
Supplement: Supplementary file 1 — Appendix S1 [file CNS-29-1907-s001.docx]

**Supplementary Material**

| Clinical variable | Q1 group (n = 49) | Q2-3 group (n = 99) | Q4 group (n = 50) |
| --- | --- | --- | --- |
| Age, years | 48.86 ± 4.31 | 62.31 ± 4.11 | 72.48 ± 2.90 |
| Sex (Male/Female) | 27/22 | 65/34 | 32/18 |
| Education, years | 15.63 ± 2.55 | 15.35 ± 2.90 | 15.20 ± 3.07 |
| Disease duration, years | 2.43 ± 3.27 | 1.76 ± 1.16 | 2.25 ± 2.98 |
| HY | 1.43 ± 0.54 | 1.71 ± 0.52 | 1.71 ± 0.46 |
| Tremor | 3.30 ± 2.98 | 4.22 ± 3.58 | 4.37 ± 3.67 |
| Rigidity | 4.02 ± 2.65 | 4.35 ± 2.89 | 4.22 ± 2.85 |
| UPDRS-III | 17.55 ± 7.76 | 22.00 ± 9.34 | 22.39 ± 10.11 |
| RBDSQ | 4.33 ± 2.75 | 3.98 ± 2.59 | 4.38 ± 2.83 |
| SCOPA-AUT | 7.54 ± 4.57 | 10.10 ± 5.62 | 10.29 ± 5.65 |
| LNS | 12.20 ± 2.61 | 10.35 ± 2.36 | 8.98 ± 2.85 |
| BJLOT | 13.24 ± 1.68 | 12.93 ± 2.02 | 11.56 ± 2.70 |
| SFT | 53.20 ± 11.91 | 50.39 ± 11.55 | 44.52 ± 10.69 |
| SDMT | 45.73 ± 10.03 | 40.16 ± 9.81 | 35.54 ± 9.75 |
| MoCA | 27.84 ± 2.44 | 26.44 ± 2.75 | 26.00 ± 2.88 |
| Immediate Recall of HVLT-R | 26.63 ± 4.60 | 24.69 ± 5.33 | 22.12 ± 5.70 |
| Caudate_R | 2.18 ± 0.66 | 1.80 ± 0.58 | 1.76 ± 0.52 |
| Caudate_L | 2.22 ± 0.63 | 1.78 ± 0.61 | 1.77 ± 0.55 |
| Putamen_R | 0.87 ± 0.36 | 0.72 ± 0.33 | 0.69 ± 0.24 |
| Putamen_L | 0.93 ± 0.41 | 0.69 ± 0.30 | 0.71 ± 0.24 |
| Striatum_R | 3.05 ± 0.96 | 2.52 ± 0.85 | 2.45 ± 0.70 |
| Striatum_L | 3.16 ± 0.98 | 2.47 ± 0.85 | 2.49 ± 0.73 |
| Bilateral Caudate | 2.20 ± 0.59 | 1.79 ± 0.57 | 1.76 ± 0.49 |
| Bilateral putamen | 0.90 ± 0.29 | 0.71 ± 0.27 | 0.70 ± 0.18 |
| Bilateral striatum | 1.55 ± 0.42 | 1.25 ± 0.39 | 1.23 ± 0.31 |
| Aβ level (pg/mL) | 946.68 ± 378.69 | 826.92 ± 356.08 | 859.13 ± 406.91 |
| α-syn level (pg/mL) | 1472.27 ± 621.21 | 1422.51 ± 725.25 | 1652.58 ± 912.12 |
| Tau level (pg/mL) | 160.72 ± 49.14 | 157.40 ± 48.07 | 190.94 ± 71.82 |
| p-tau level (pg/mL) | 13.91 ± 4.76 | 13.98 ± 4.60 | 16.86 ± 6.42 |

**TABLE S1. The demographic and clinical data for each age quartile group**

The data were shown as the mean ± standard deviation (SD). The motor function examination was assessed in ON state. Abbreviations: Aβ, β-amyloid; α-syn, α-synuclein; HY, Hoehn & Yahr stage; UPDRS-III, Unified Parkinson’ s Disease Rating Scale Part III; RBDSQ, REM Sleep Behavior Disorder Screening Questionnaire; SCOPA-AUT, Scale for Outcomes in Parkinson's Disease-Autonomic; SDMT, Symbol Digit Modalities Test; LNS, Letter Number Sequencing; SFT, Semantic Fluency Test Score; BJLOT, Benton Judgement of Line Orientation; MoCA, Montreal Cognitive Assessment; HVLT-R, Hopkins Verbal Learning Test – Revised; SBR, striatal binding ratio; CSF, cerebrospinal fluid.

| Group difference of power FD values among different age quartile groups | | | | |
| --- | --- | --- | --- | --- |
| Group  Metric | **Q1 group**  **(n = 18)** | **Q2-3 group**  **(n = 37)** | **Q4 group**  **(n = 19)** | ***p* value** |
| Power FD values | 0.29 ± 0.17 | 0.24 ± 0.11 | 0.29 ± 0.15 | *p* > 0.05 |
|  |  |  |  |  |
| Group difference of power FD values between male and female patients | | | | |
| Group  Metric | **Male patients**  **(n =48)** | **Female patients**  **(n = 26)** | ***p* value** | |
| Power FD values | 0.27 ± 0.13 | 0.26 ± 0.15 | *p* > 0.05 | |

**TABLE S2. Group differences of power FD values**

The data were shown as the mean ± standard deviation (SD). One-way ANOVA test was used to compare power FD values among 3 age quartile groups. The unpaired t-test was used to compare power FD values between male and female patients. Abbreviations: FD, frame-wise displacement.


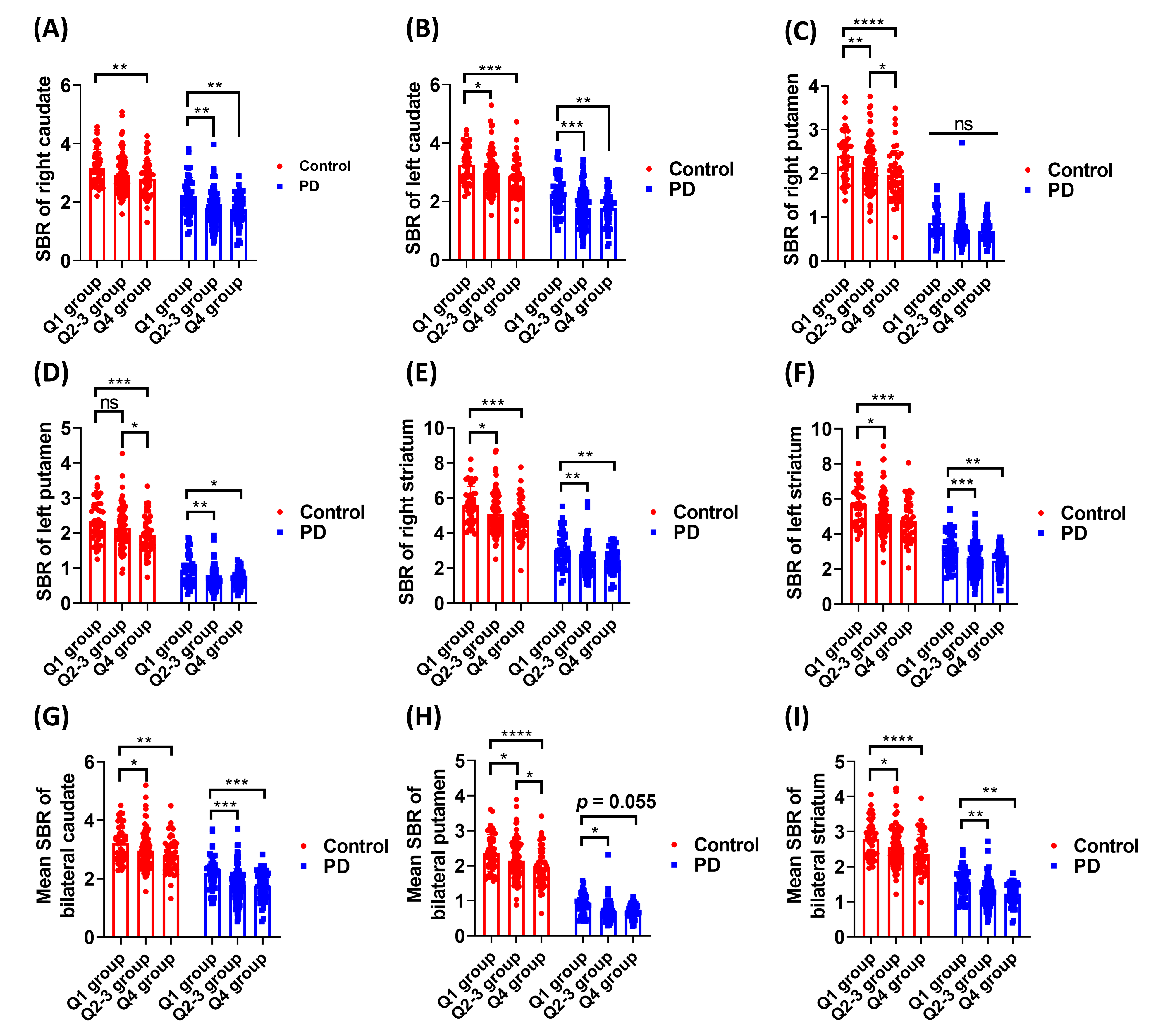


**FIGURE S1.** Group differences of striatum SBRs among different age quartiles of control and PD participants. Group differences of SBRs in right caudate (A), left caudate (B), right putamen (C), left putamen (D), right striatum (E), left striatum (F), bilateral caudate (G), bilateral putamen (H), and bilateral striatum (I) among different age quartiles of control and PD participants. Two-way ANOVA test followed by Tukey’s post-hoc test was used for the comparisons of striatum SBRs among different age quartiles of control and PD participants. **p* < 0.05, ***p* < 0.01, ****p* < 0.001, *****p* < 0.0001. Abbreviations: SBR, striatal binding ratio.


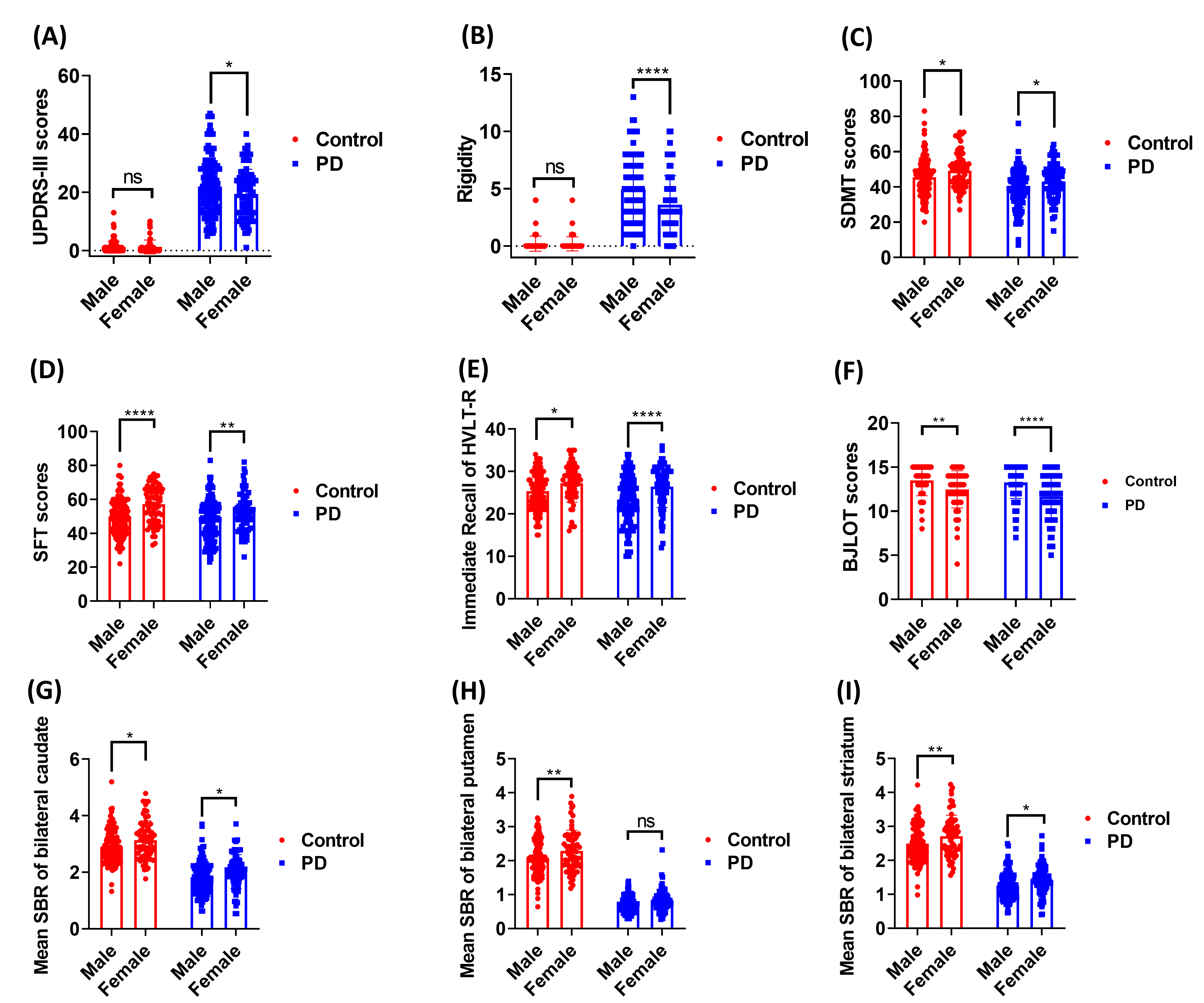


**FIGURE S2.** Group differences of clinical variables between male and female participants. Group differences of scores of UPDRS-III (A), rigidity (B), SDMT (C), SFT (D), Immediate Recall of HVLT-R (E), and BJLOT scores (F) between female and male participants. Group differences of SBRs in bilateral caudate (G), bilateral putamen (H), and bilateral striatum (I) between female and male participants. Two-way ANOVA test followed by Tukey’s post-hoc test was used for the comparisons of clinical variables between male and female participants. **p* < 0.05, ***p* < 0.01, ****p* < 0.001, *****p* < 0.0001. Abbreviations: UPDRS-III, Unified Parkinson’ s Disease Rating Scale Part III; SDMT, Symbol Digit Modalities Test; SFT, Semantic Fluency Test Score; HVLT-R, Hopkins Verbal Learning Test – Revised; BJLOT, Benton Judgement of Line Orientation; SBR, striatal binding ratio.


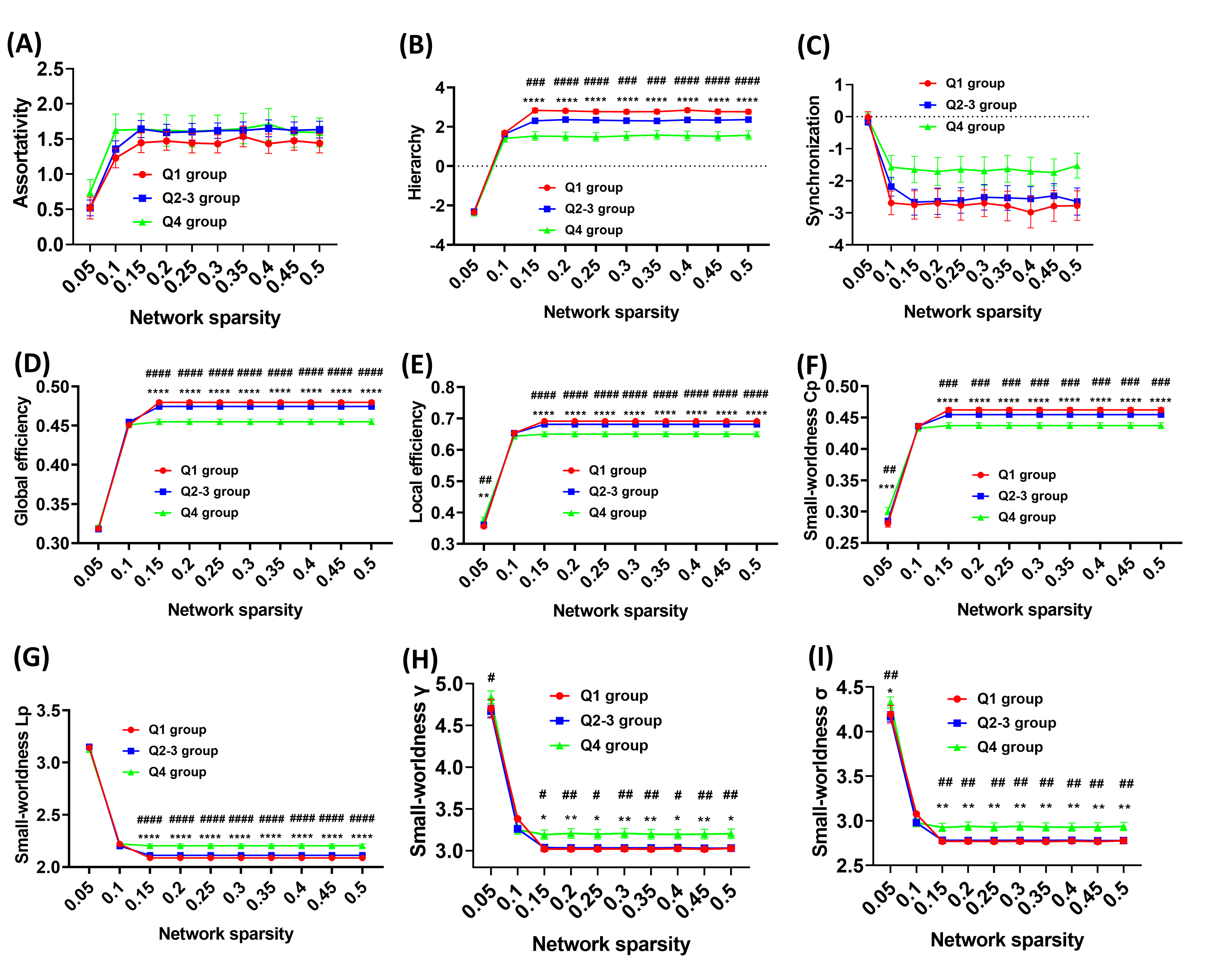


**FIGURE S3.** Group differences of white matter network metrics in multiple sparsity threshold among different age quartiles of PD patients. Group differences of assortativity (A), hierarchy (B), synchronization (C), global efficiency (D), local efficiency (E), small-worldness Cp (F), small-worldness Lp (G), small-worldness γ (H), small-worldness σ (I). Two-way ANOVA test followed by FDR correction was used for the comparisons of graphical metrics among three age quartile groups. Q1 group *vs* Q4 group: **p* < 0.05, ***p* < 0.01, ****p* < 0.001, *****p* < 0.0001. Q2-3 *vs* Q4 group: ^#^*p* < 0.05, ^##^*p* < 0.01, ^###^*p* < 0.001, ^####^*p* < 0.0001. Abbreviations: Cp, clustering coefficient; Lp, characteristic path length.


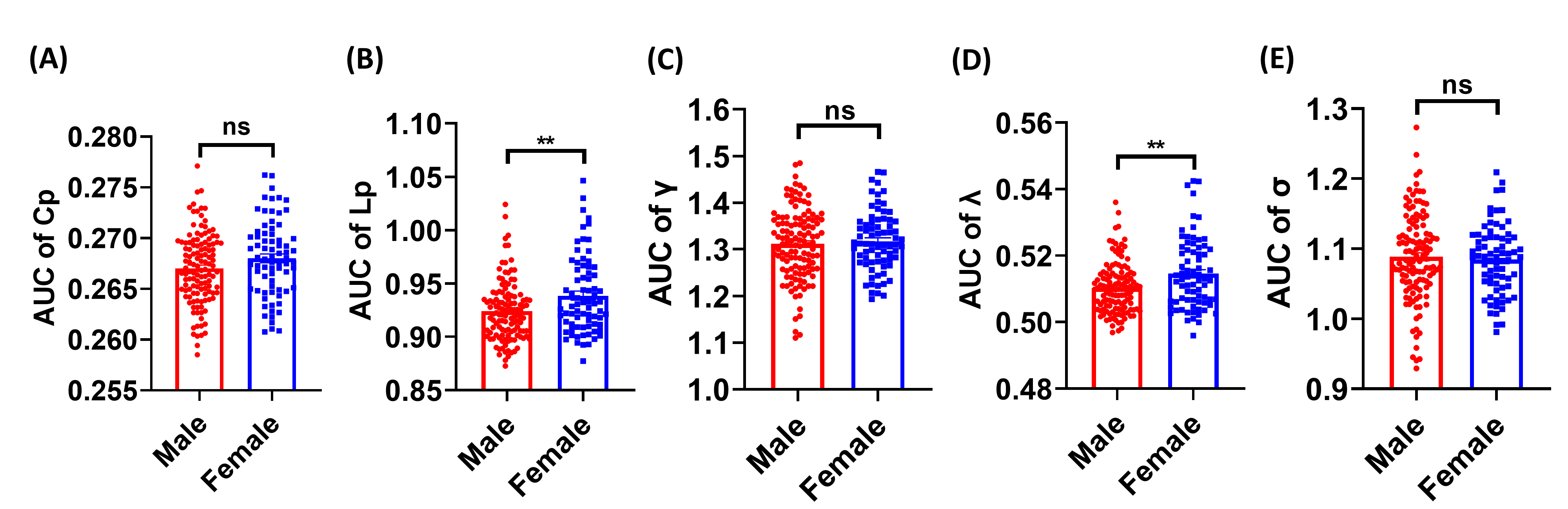


**FIGURE S4.** Group differences of small-worldness properties of gray matter covariance network between male and female patients. Group differences in the AUC of Cp (A), AUC of Lp (B), AUC of γ (C), AUC of λ (D), and AUC of σ (E) between male and female patients. Unpaired t-test followed by FDR correction was used for the comparisons of AUC of graphical metrics between male and female patients. Abbreviations: AUC, area under curve; Cp, clustering coefficient; Lp, characteristic path length.


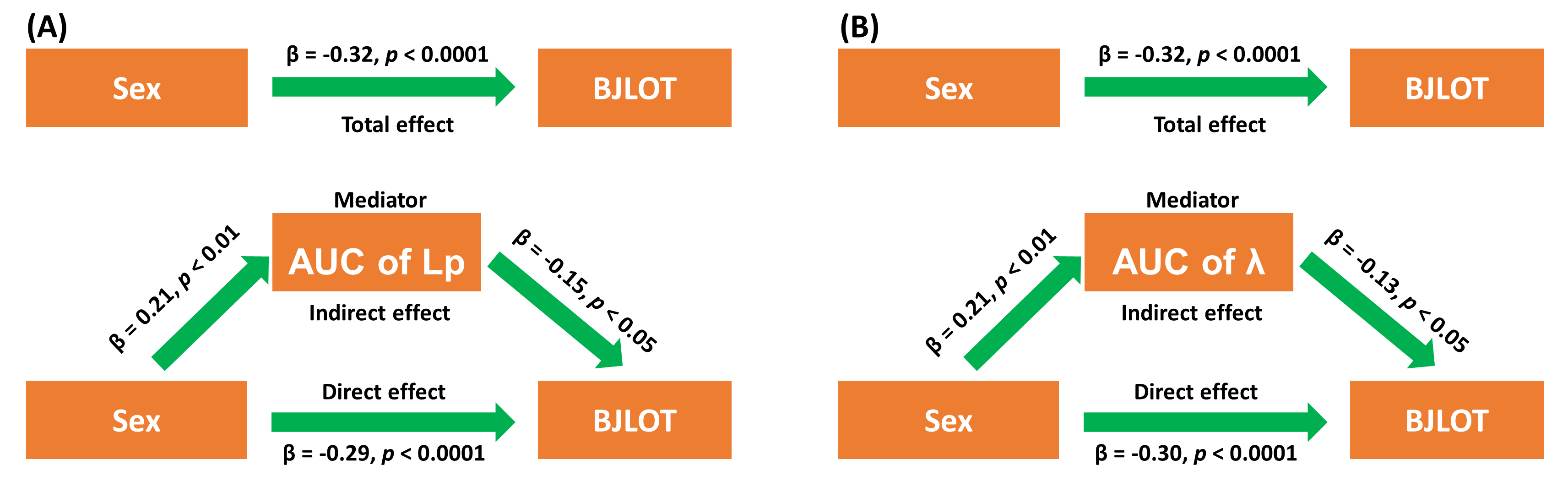


**FIGURE S5.** The small-worldness properties in gray matter covariance network mediated the effects of sex on BJLOT scores of PD patients. Mediation analysis of AUC of Lp and BJLOT score (A), AUC of λ and BJLOT score (B). During the mediation analysis, age, years of education, and disease duration were included as covariates. *p* < 0.05 was considered statistically significant. Abbreviations: AUC, area under curve; BJLOT, Benton Judgement of Line Orientation; Lp, characteristic path length.
